# Exploring Eye, Hair, and Skin Pigmentation in a Spanish Population: Insights from Hirisplex-S Predictions

**DOI:** 10.3390/genes15101330

**Published:** 2024-10-16

**Authors:** Belén Navarro-López, Miriam Baeta, Victoria Suárez-Ulloa, Rubén Martos-Fernández, Olatz Moreno-López, Begoña Martínez-Jarreta, Susana Jiménez, Iñigo Olalde, Marian M. de Pancorbo

**Affiliations:** 1 BIOMICs Research Group, Lascaray Research Center, University of the Basque Country UPV/EHU, 01006 Vitoria-Gasteiz, Spain; belennavarrolopez1997@gmail.com (B.N.-L.);; 2 Department of Zoology and Animal Cellular Biology, Faculty of Pharmacy, University of the Basque Country UPV/EHU, 01006 Vitoria-Gasteiz, Spain; 3 Bioaraba Health Research Institute, 01009 Vitoria-Gasteiz, Spain; 4 Panacea Cooperative Research S. Coop, 24001 Castilla y León, Spain; 5 Department of Legal Medicine, Toxicology, and Physical Anthropology, University of Granada, 18071 Granada, Spain; 6 Department of Physical Anthropology, Society of Sciences Aranzadi, 20014 Donostia, Spain; 7 Faculty of Medicine, University of Zaragoza, 50009 Zaragoza, Spain; 8 Aragon Health Research Institute (IIS-Aragón), 50009 Zaragoza, Spain; 9 Department of Pathology and Surgery, University of Miguel Hernández, 03550 Alicante, Spain; 10 Ikerbasque-Basque Foundation of Science, 48009 Bilbao, Spain

**Keywords:** FDP, eyes, hair, and skin pigmentation, prediction model, Spanish population

## Abstract

Background/Objectives: Understanding and predicting human pigmentation traits is crucial for individual identification. Genome-wide association studies have revealed numerous pigmentation-associated SNPs, indicating genetic overlap among pigmentation traits and offering the potential to develop predictive models without the need for analyzing large numbers of SNPs. Methods: In this study, we assessed the performance of the HIrisPlex-S system, which predicts eye, hair, and skin color, on 412 individuals from the Spanish population. Model performance was calculated using metrics including accuracy, area under the curve, sensitivity, specificity, and positive and negative predictive value. Results: Our results showed high prediction accuracies (70% to 97%) for blue and brown eyes, brown hair, and intermediate skin. However, challenges arose with the remaining categories. The model had difficulty distinguishing between intermediate eye colors and similar shades of hair and exhibited a significant percentage of individuals with incorrectly predicted dark and pale skin, emphasizing the importance of careful interpretation of final predictions. Future studies considering quantitative pigmentation may achieve more accurate predictions by not relying on categories. Furthermore, our findings suggested that not all previously established SNPs showed a significant association with pigmentation in our population. For instance, the number of markers used for eye color prediction could be reduced to four while still maintaining reasonable predictive accuracy within our population. Conclusions: Overall, our results suggest that it may be possible to reduce the number of SNPs used in some cases without compromising accuracy. However, further validation in larger and more diverse populations is essential to draw firm conclusions and make broader generalizations.

## 1. Introduction

The recovery of genetic profiles based on short tandem repeat markers (STRs) and single nucleotide polymorphisms (SNPs) is crucial in Forensic Sciences for individual identification. By comparing the genetic profiles of known suspects with evidence, cases can be solved. However, cold cases occur when no match is found. Recent advances have led to Forensic DNA Phenotyping (FDP), which predicts Externally Visible Characteristics (EVCs) from the DNA of an unknown individual. This aids identification investigations by narrowing down not only suspects but also missing persons and disaster victims. To date, human pigmentation, especially iris, hair, and skin pigmentation, is one of the most studied EVCs [1,2,3].

Genome-wide association studies (GWAS) have uncovered numerous pigmentation-associated SNPs, showing significant genetic overlap among pigmentation traits. This has sparked interest in developing predictive models without the need to study large numbers of SNPs. Different SNP panels for predicting pigmentation phenotype have been developed. However, HIrisPlex-S remains the most widely used FDP tool so far [4,5,6,7,8,9,10].

Since 2007, when the first two DNA-based eye color prediction studies were conducted [3,11], numerous investigations have been carried out in this area. SNPs within various genes, including *ASIP*, *DSCR9*, *HERC2*, *IRF4*, *KITLG*, *LYST*, *MC1R*, *MYO5A*, *NPLOC4*, *OCA2*, *SLC24A4*, *SLC24A5*, *TYR*, and *TYRP1*, have been used for DNA prediction of iris pigmentation [3,4,6,7,10,11,12,13,14,15,16,17,18,19,20,21]. However, the first comprehensive DNA prediction study on eye color established a minimal set of six SNPs from six genes for iris pigmentation prediction (*HERC2* rs12913832, *OCA2* rs1800407, *SLC24A4* rs12896399, *SLC45A2* rs16891982, *TYR* rs1393350, and *IRF4* rs12203592) [13]. This set forms the basis for the IrisPlex system, the first DNA-based eye color prediction system for forensic purposes, providing probabilities for blue, brown, and intermediate eyes [10,22]. Subsequent studies have identified additional SNPs, some overlapping and some in linkage disequilibrium, with SNPs present in IrisPlex. This has led to increased accuracy, although it depends on the population examined [4,6,15,16,17,19,23,24].

In 2013, the IrisPlex system expanded its scope beyond eye color to include hair color prediction, leading to the creation of the HIrisPlex system. This model comprises a total of 24 SNPs, including the original six from IrisPlex and 18 additional SNPs associated with hair pigmentation (*MC1R* Y152OCH, *MC1R* N29insA, *MC1R* rs1805006, *MC1R* rs11547464, *MC1R* rs1805007, *MC1R* rs1805008, *MC1R* rs1805009, *MC1R* rs1805005, *MC1R* rs2228479, *MC1R* rs1110400, *MC1R* rs885479, *SLC45A2* rs28777, *SLC45A2* rs16891982, *KITLG* rs12821256, *EXOC2* rs4959270, *IRF4* rs12203592, *TYR* rs1042602, *TYR* rs1393350, *OCA2* rs1800407, *SLC24A4* rs2402130, *SLC24A4* rs12896399, *HERC2* rs12913832, *ASIP/PIGU* rs2378249, and *TYRP1* rs683) [8]. It is noteworthy that the initial DNA-based hair prediction efforts were limited to red hair [25]. Although an attempt was made in 2007 to predict all hair pigmentation categories from DNA, the accuracy for non-red hair colors was notably low [11]. It was not until the development of HIrisPlex that, thanks to the subsequent discoveries, the first DNA system capable of accurately predicting four different hair color categories (blond, red, brown, and black) was established.

Genetic knowledge about skin pigmentation is currently much less available because of the requirement of a more diverse global population for analyses [1]. Unlike iris or hair color prediction, the initial system for predicting DNA skin color took longer to develop. Maroñas et al. first developed the system in 2014, and Chaitanya et al. used it to expand the HIrisPlex system to the HIrisPlex-S, consisting of 41 SNPs, 36 of which are predictive of skin color [5,9]. Another distinction between eye and hair prediction models is that, although research continues in these areas, advancements do not seem to offer significant advantages, with the HIrisPlex a quite comprehensive predictive system. However, the genetic understanding of skin pigmentation is still evolving [1,26]. Research into genetic loci associated with sun sensitivity as an indirect measure of skin color, as well as markers related to skin color in non-European populations, has led to the discovery of new SNPs that could greatly improve prediction accuracies with current systems [27,28,29].

While the high accuracy rates of the HIrisPlex-S are quite encouraging, there are several areas of concern regarding the use of categories to classify individuals, especially within the intermediate spectrum between categories [1,26]. Although this predictive model was developed and validated using various European populations, the main objective of this study was to estimate its accuracy when applied exclusively to a specific population, in this case, the Spanish population, in order to evaluate its ability to predict all categories. In addition, the specific SNP set for this population has been refined with the purpose of obtaining more accurate values in its individuals’ pigmentation prediction.

## 2. Materials and Methods

### 2.1. Sample Collection

This research involved 412 individuals from the Spanish population. Ethical approval (M10_2021_143) was provided by the Ethics Committee for Research on Human Subjects of the University of the Basque Country, CEISH-UPV/EHU, BOPV 32, 17/2/2014. The study included individuals of diverse sexes and legal ages, of European ancestry, and belonging to the Spanish population. Recruitment was conducted across a range of academic centers, including universities, vocational training centers, and secondary schools, located in different regions of Spain, specifically, the Basque Country, Zaragoza, Alicante, and Granada. Additionally, the inherent mobility among students means that our sample also includes individuals from other regions of Spain, encompassing the north, south, east, west, and central areas. Students from regions outside the sampling locations represented a minority, which may explain the underrepresentation of certain categories in the study.

All participants gave written informed consent prior to participation. Each volunteer provided saliva samples by sterile swabs in triplicate, and a facial 3D scan was performed using a portable white light-led 3D scanner Academia 3D/20 (Creaform, Levis, QC, Canada) following the protocols published by the 3D Facial Norms (3DFN) Project [30,31] and Heike et al. [32].

Additionally, participants completed a questionnaire that included basic data such as sex, age, and information on eye and hair pigmentation phenotypes. Eye and hair color data were classified using the categories of the HIrisPlex-S system: blue, intermediate (specifying color), and brown for eye color, and blond, red, brown, and black for hair color [9]. In 40 of the participants, a validation of the provided data was conducted by a researcher who also assigned the eye and hair color. In all cases, there was agreement, suggesting that the self-reported information was sufficiently accurate. Three independent researchers assigned each volunteer’s skin color into the five categories proposed by the HIrisPlex-S system: very pale, pale, intermediate, dark, and dark black [9]. When unanimity was not achieved, an additional researcher was involved. If a 3-to-1 consensus still could not be reached, skin pigmentation was not assigned for that individual.

### 2.2. DNA Extraction and Genotyping

DNA from saliva swabs was isolated by the salting out method [33] using the DNA Purification System PuregenTM (Gentra System, Inc., Minneapolis, MN, USA). Quantity as well as the quality of the DNA obtained after extraction was evaluated by spectrophotometry with a NanoDrop™ One (Thermofisher Scientific, Waltham, MA, USA) and fluorimetry with a Qubit^®^ 2.0 Fluorometer and the Qubit^®^ dsDNA HS Assay Kit, 0.1–120 ng (Thermofisher Scientific). Once quantified, DNAs were diluted in Milli-Q water to 10–60 ng/μL for Fluidigm analysis (Fluidigm Corp., South San Francisco, CA, USA), as well as to 1 ng/uL for SNaPshot minisequencing analysis (Applied Biosystems, Foster City, CA, USA) and stored at −20 °C.

Genotyping analysis was conducted for the 41 SNPs of the HIrisPlex-S system, which includes 17 predictive SNPs for skin color and 24 predictive SNPs for eye and hair color, with 19 SNPs contributing to both skin and eye/hair color prediction [9]. These 41 SNPs were analyzed in a larger panel of 157 markers related to various EVCs, including pigmentation traits. The additional SNPs in this panel were examined for purposes outside of the scope of this study, which focuses solely on the 41 pigmentation-related SNPs. Therefore, pre-existent panels specifically developed for the HIrisPlex-S system were not employed in this analysis. Due to the need for simultaneous analysis of a large number of SNPs, Fluidigm technology was selected as the primary genotyping method. Although all 41 pigmentation SNPs underwent evaluation for Fluidigm technology analysis, only 31 were suitable for this methodology. Thus, these 31 SNPs (Supplementary Table S1) were genotyped employing one Juno 96 × 96 and two 48.48 Fluidigm SNPtypes genotyping systems (Fluidigm Corp.). For the remaining 10 SNPs (Supplementary Table S1), there were observed incompatibilities with this technique. Therefore, these 10 SNPs, along with other EVC genetic markers that exhibited similar difficulties, were analyzed using SNaPshot minisequencing technology.

SNaPshot analysis-Primer design. Primer design, as well as melting temperature, GC% content, and the possible formation of hairpin and dimers on each pair of primers checking was carried out using PerlPrimer v1.1.21 software [34]. Web-based AutoDimer software v1.0 [35] was used to study the compatibility of primers in multiplex reactions. Finally, specificity was evaluated with primer-BLAST [36]. Primers used in amplification range in size from 114 to 241 bp. Attempts were made in all cases to select primers that produced the shortest possible amplicons so that they could be used in samples with high limitations in their DNA content and/or moderate DNA degradation levels (Supplementary Table S2).

Minisequencing primers were designed manually to hybridize the adjacent region of the SNP. Nevertheless, melting temperatures, as well as potential unfavorable reactions between them, were also studied with the web-based AutoDimer software [35]. These primers were augmented with a 10- to 65-bp non-binding tail at the 5′ end in order to ensure various sizes of the minisequencing products, with a minimum difference of 5 bp (Supplementary Table S2).

SNaPshot analysis-Multiplex PCR amplification. PCR multiplex amplification was carried out as follows: 5 μL of Multiplex PCR Master Mix (QIAGEN, Hilden, Germany), 1 μL of primermix (final concentration of each primer shown in Supplementary Table S2), 2 ng of genomic DNA and Milli-Q water in order to reach a final reaction volume of 10 μL. The amplification was performed in a C1000TM Thermal Cycler (BioRad, Hercules, CA, USA) under the following conditions: initial denaturalization at 95 °C for 15 min; 35 cycles at 95 °C for 30 s, 60 °C for 50 s, and 65 °C for 40 s; and a final extension of 6 min at 65 °C.

Amplified DNA was treated with ExoSAP (Takara Bio Inc., Shiga, Japan) to eliminate the remaining primers and nucleotides: 1 μL of ExoSAP per 2.5 μL of PCR product, incubated at 37 °C for 5 min followed by enzymatic inactivation at 80 °C for 1 min.

SNaPshot analysis-Minisequencing and capillary electrophoresis. Minisequencing was carried out in a final volume of 7 μL containing 2 μL of SNaPshot™ Multiplex Kit reaction mix (Applied Biosystems, Foster City, CA, USA), 1 μL of primermix (final concentration of each primer shown in Supplementary Table S2), 3 μL of Milli-Q water and 1 μL of purified multiplex PCR product. Thermocycling conditions in a C1000™ Thermal Cycler (BioRad) were 25 cycles at 96 °C for 10 s, 55 °C for 5 s, and 60 °C for 30 s.

Minisequencing products were further purified by enzymatic digestion adding 1 μL of SAP (Takara Bio Inc.) to 2 μL of product and incubated at 37 °C for 60 min to remove any remaining nucleotides, followed by enzyme denaturation at 80 °C for 15 min.

Finally, minisequencing products were analyzed by mixing 1 μL of purified product, 12 μL of Hi-DI formamide (Applied Biosystems), and 0.38 μL of Gene-Scan 120LIZ (Applied Biosystems). After denaturation, capillary electrophoresis was conducted in an ABI PRISM^®^ 3130 Genetic Analyzer (Applied Biosystems). Data were analyzed using GeneMapper^®^ Software v4.0 (Applied Biosystems).

### 2.3. Statistical Analysis

Population genetic parameters. Allele and genotype frequencies were calculated using Arlequin v3.5.2.2 [37]. Differences in population parameters were assessed using a Chi-square test (χ2) conducted with IBM SPSS Statistics v28 software [38] and taking as reference previously published data from European and Iberian populations [39], as these are the most closely related to the Spanish population under study. Hardy-Weinberg Equilibrium (HWE) was also analyzed using Arlequin v3.5.2.2 software. *p*-value threshold was set after Bonferroni correction (α = 0.05/37 = 1.314 × 10^−3^).

Pigmentation prediction. Eye, hair, and skin pigmentation were predicted from DNA using the online HIrisPlex-S tool, available via the Erasmus MC Hirisplex website (https://hirisplex.erasmusmc.nl/, accessed on 24 July 2024). This tool calculates prediction probabilities for iris (blue, intermediate, and brown), hair (blond, red, brown, and black), and skin color (very pale, pale, intermediate, dark, and dark-to-black) using the multinomial logistic regression (MLR) model. Predicted phenotypes were compared with the actual data to assess precision. The overall prediction performance of each model was evaluated by calculating the accuracy, the area under the curve (AUC), the sensitivity, the specificity, and the positive and negative predictive value (PPV and NPV) using an R script. These metrics were calculated according to Liu et al. [13], utilizing two-by-two contingency tables of predicted and observed color types.

Furthermore, a model for each phenotypic character was calculated based on the genotypic and phenotypic data from our collection using MLR analysis. The DNA-based prediction models were developed through the forward selection of genetic variants based on the Akaike information criterion (AIC), with the optimal model being the one with the smallest AIC value, balancing goodness-of-fit and parsimony. The forward selection process begins with an empty model and gradually adds predictor variables. At each iteration, the variable that provides the greatest improvement to the model is included. This model-building process continues until no further improvement is observed by adding additional variables, ensuring that all included SNPs contribute significantly and minimizing estimated information loss. This methodology allowed us to evaluate the significance level of the present SNPs in the HIrisPlex-S system in our Spanish population and identify the most relevant ones for our specific population. To evaluate the predictive ability of the models, a repeated 10-fold cross-validation approach was applied. These analyses were conducted using custom programs written in R and utilizing the ‘caret’ package.

## 3. Results and Discussion

Genotypic results were obtained for all SNPs except for rs1800414, rs2228479, rs312262906, and rs6497292. None of these four markers were essential for pigmentation prediction [9], and, in addition, they exhibited limited variability within populations. Specifically, in the European population, rs312262906 and rs1800414 demonstrated a minor allele frequency (MAF) < 0.01, whereas rs2228479 and rs6497292 had an MAF < 0.09 (Supplementary Table S1). Consequently, it was decided to proceed with the remaining 37 markers for population genetic parameters and pigmentation prediction analysis.

### 3.1. Population Genetic Parameters

Allele and genotype frequencies are presented in Supplementary Table S3. Allele frequencies in our population sample were compared to those previously published for the Iberian population [39] to assess representativeness. No statistically significant differences were found. Furthermore, no departure from HWE was observed, except for rs1667394, rs2238289, and rs4959270, showing a slight excess of heterozygotes (Supplementary Table S3).

### 3.2. Pigmentation Prediction

**Figure 1:**
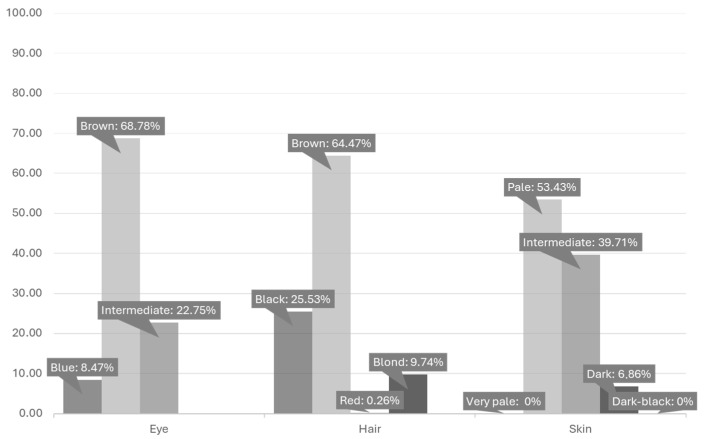
Distribution of eye, hair, and skin pigmentation categories in our sample collection.

**Figure 2:**
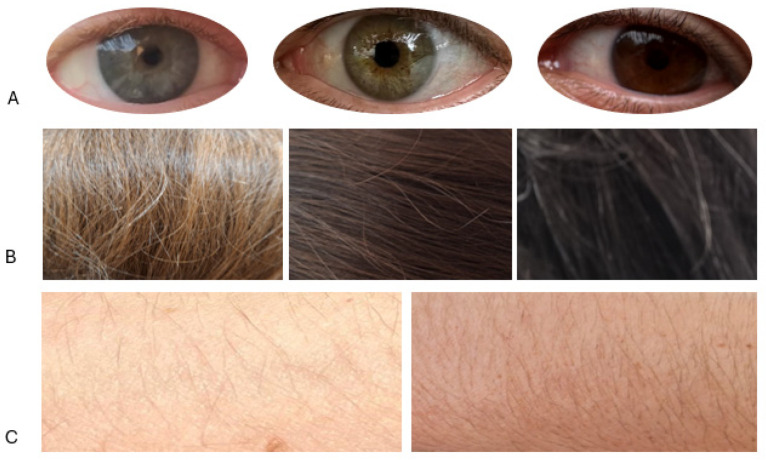
Real examples of eye (**A**), hair (**B**), and skin (**C**) pigmentation represent our main categories (with a representation greater than 8%). (**A**) From left to right: blue, intermediate, and brown eyes. (**B**) From left to right: blond, brown, and black hair. (**C**) From left to right: pale and intermediate skin.

Out of the initial sample set comprising 412 individuals, there was a final count of 378 for eye pigmentation, 380 individuals for hair pigmentation, and 408 for skin pigmentation due to incomplete data (Figure 1 and Figure 2).

#### 3.2.1. Eye Pigmentation

As previously mentioned, iris pigmentation data were gathered using the three categories proposed by the HIrisPlex-S system (blue, intermediate, and brown) [9]. Blue-green, blue-brown, brown-green, brown-blue-green, green, grey, hazel, and light eyes were grouped into the intermediate category. Analysis of the distribution of eye pigmentation (Figure 1 and Figure 2) showed a majority of individuals with brown eyes (68.78%), followed by intermediate (22.75%) and blue eyes (8.47%).

For iris pigmentation prediction, the six SNPs specifically established by the HIrisPlex-S for eye color (*HERC2* rs12913832, *OCA2* rs1800407, *SLC24A4* rs12896399, *SLC45A2* rs16891982, *TYR* rs1393350, and *IRF4* rs12203592) were used. Two individuals could not be assigned because of incomplete genetic profiles and a lack of data for rs12913832.

To determine the final phenotype prediction from DNA using probabilities derived from the HIrisPlex-S tool, an initial threshold of 0.7 was set, as proposed by Liu et al. [13]. While a probability value > 0.7 indicated a positive prediction, values ranging between 0.5 and 0.7 were categorized as undefined, potentially representing a combination of the blue or brown category (depending on which had the highest probability) and an intermediate phenotype, as described in Walsh et al. [40]. Under the initial conditions, no individuals were classified as having an intermediate eye color. Therefore, to allow for greater flexibility within this category, cases where the probability values for both blue and brown eye predictions were close and neither exceeded the 0.5 thresholds were classified as intermediate. These predictions were compared to the actual data to assess model accuracy, considering correct, incorrect, or uncertain matches (Supplementary Table S4). A correct match was defined when both the predicted and actual data were the same; an incorrect match was assigned when the predictive color was definitive and differed from the actual data; and an uncertain match was considered when the predicted color was undefined, as it remained unclear whether the match was accurate (Table 1). This method will hereafter be referred to as the strict approach.

Given the high percentage of uncertain matches observed in certain categories and to simplify the outcomes into correct or incorrect matches, a more flexible approach was followed. Similar to the strict method, a probability value exceeding 0.7 was considered a positive prediction for the blue and brown categories, and cases where the probability values for both blue and brown eye predictions were close, with neither exceeding the 0.5 threshold, were classified as intermediate. However, in this flexible approach, values ranging between 0.5 and 0.7, previously categorized as undefined, were now classified as intermediate-blue or intermediate-brown, depending on whether the blue or brown category had a higher probability (Supplementary Table S4). Consequently, all undefined predictions were reclassified as either intermediate-blue or intermediate-brown. Uncertain matches were then reassigned as correct if one of the two predicted colors (intermediate and blue or brown) matched the actual color and as incorrect if neither of the two predicted colors corresponded with the actual color (Table 2).

The HIrisPlex-S system has demonstrated accurate prediction of blue and brown eye color, while its predictive capacity for intermediate eye colors has been pointed out as a big limitation [1,10]. This is clearly reflected in our results, where we observed a prediction accuracy of 93.75% for blue eyes and a range between 88.08 to 96.92%, depending on the applied approach, for brown eyes (Table 1 and Table 2). However, there was a high rate of incorrect assignment for intermediate eyes predictions (66.28%) regardless of the method used. Interestingly, the majority of intermediate eyes (51.16%) were misclassified as brown by the HIrisPlex-S tool. Although the total number of incorrect cases remained unchanged, the flexible approach led to an increase in correct predictions. Specifically, 23.26% of cases that were strictly unclassified were reassigned as correct under the second approach, thereby raising the percentage of correct matches from 10.47% (Table 1) to 33.72% (Table 2). Nevertheless, even with the application of more flexible criteria, the majority of intermediate eye colors continued to be inaccurately classified.

Given the improved outcomes with the flexible approach, the overall prediction performance metrics of the model were calculated based on this method. While high sensitivity values were observed for both blue (93.75%) and brown (97.67%) eyes, specificity was notably high for blue pigmentation (95.93%) but lower for brown eyes (62.71%) (Table 3). This discrepancy can be attributed to the large number of individuals with intermediate eyes who were predominantly misclassified as brown. For the intermediate pigmentation prediction, sensitivity was low (33.72%), while specificity remained high (97.59%). This indicates that although intermediate cases are frequently misclassified when the model does predict intermediate pigmentation, it is highly likely to be accurate. The AUC values obtained were 0.948 for blue, 0.802 for brown, and 0.344 for intermediate, lower compared to those of the IrisPlex model, which are 0.94, 0.95, and 0.74 for blue, brown, and intermediate eyes, respectively [10,13]. Overall, the results obtained align with those observed in other populations [41,42].

Our low performance for the intermediate category is consistent with previous studies, which have also reported a high percentage of false predictions for intermediate eye colors [24,41,42,43]. Moreover, they also align with what Kayser stated [1], that since blue and brown eyes are the two extreme categories, and the intermediate ones represent the continuum between them, predicting the intermediate category of iris pigmentation is more challenging than for blue and brown. As previously mentioned, although the application of more flexible criteria did improve the rate of correct matches in our study, the majority of intermediate eyes were still inaccurately classified. It is also important to note that the intermediate category showed a diverse range of subcategories from blue-green to grey to hazel. While the distinctions between brown and blue eyes are clear, the complexity within the intermediate spectrum introduces a level of subjectivity that may contribute to inaccuracies when distinguishing between intermediate and blue, as well as between intermediate and brown in certain cases. Therefore, future approaches should consider extending DNA prediction of eye pigmentation from categorical to continuous to minimize the interpretative challenges associated with current categorical results [1,26].

The first DNA-based iris pigmentation prediction system for forensic purposes, the IrisPlex system, was based on a minimal set of six SNPs (*HERC2* rs12913832, *OCA2* rs1800407, *SLC24A4* rs12896399, *SLC45A2* rs16891982, *TYR* rs1393350, and *IRF4* rs12203592) [10,13]. Subsequent studies have identified additional SNPs, some overlapping with or in linkage disequilibrium with SNPs present in IrisPlex. This has led to increased performance, although it varies depending on the population examined [4,6,15,16,17,19,23,24]. Studies differ on which SNPs are truly essential for accurate eye pigmentation prediction; some argue for increasing the number of markers analyzed [6], others find the original six from IrisPlex adequate [44], and yet others propose reducing the number of markers [45]. With these perspectives in mind and given that the intermediate category remained largely misclassified despite the adoption of a more flexible approach, we recalculated the IrisPlex model using the same SNPs as input predictor variables but tailoring them specifically to our population.

**Figure 3:**
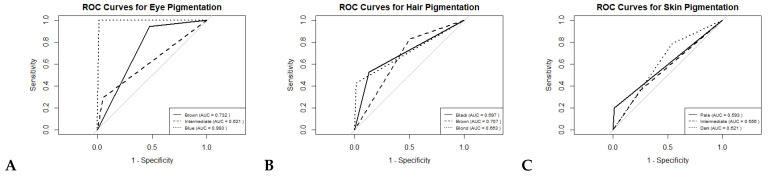
ROC curves for eye (**A**), hair (**B**), and skin (**C**) pigmentation prediction models.

The forward stepwise MLR produced a model based on four of the six SNPs (rs12913832, rs16891982, rs1800407, and rs12203592), suggesting that these genetic markers are the most informative for predicting iris pigmentation within our population. For our sample group, these SNPs showed a reasonable capacity to predict eye color. It is noteworthy that the marker *HERC2* rs12913832 showed the highest association, consistent with previous studies highlighting this marker as the most associated with eye color [16,24,45,46,47]. After cross-validation, the AUC values for predicting each eye pigmentation were 0.993 for blue, 0.732 for brown, and 0.621 for intermediate (Figure 3). Overall, our findings remain slightly below those reported by the IrisPlex model (AUC = 0.94 for blue, AUC = 0.95 for brown, and AUC = 0.74 for intermediate eyes) [10,13], and they are relatively consistent with the results obtained using the IrisPlex model on the same population (Table 3). Differences could arise from differences in sample sizes, as IrisPlex is now based on close to 9500 samples [26], as well as the uneven distribution of categories in our dataset. However, these outcomes show a considerable improvement in the prediction performance for intermediate eyes compared to previous results from our population using the IrisPlex model (Table 3).

In summary, SNPs *HERC2* rs12913832, *SLC45A2* rs16891982, *OCA2* rs1800407, and *IRF4* rs12203592 were the most informative for estimating iris color in our population. When predictions were repeated using the online HIrisPlex-S tool with only the data from these four markers, the results remained largely unchanged. The accuracy for blue and intermediate eye color categories stayed consistent at 93.75% and 33.72% correct matches, respectively, while a slight decrease was observed for brown eyes, from 96.92% to 94.23%. However, these findings suggest that reducing the number of markers can still yield adequate prediction performance, which could be particularly useful for cases of highly degraded DNA. Nevertheless, these results were observed in a relatively small population, and further validation in larger and more diverse populations is needed before drawing definitive conclusions.

#### 3.2.2. Hair Pigmentation

Hair pigmentation data were collected using the four categories proposed by the HIrisPlex-S system (blond, red, brown, and black) [9]. Upon observing the distribution of hair color (Figure 1 and Figure 2), a predominance of individuals fell into the brown hair category (64.47%), followed by black (25.53%), blond (9.74%), and red hair (0.26%).

For hair pigmentation prediction, 20 out of the 22 SNPs specifically established by the HIrisPlex-S for hair color (*MC1R* rs201326893, *MC1R* rs1805006, *MC1R* rs11547464, *MC1R* rs1805007, *MC1R* rs1805008, *MC1R* rs1805009, *MC1R* rs1805005, *MC1R* rs1110400, *MC1R* rs885479, *SLC45A2* rs28777, *SLC45A2* rs16891982, *KITLG* rs12821256, *EXOC2* rs4959270, *IRF4* rs12203592, *TYR* rs1042602, *OCA2* rs1800407, *SLC24A4* rs2402130, *HERC2* rs12913832, *ASIP/PIGU* rs2378249, and *TYRP1* rs683) were used. The HIrisPlex prediction guide for interpreting individual hair color probabilities was followed to determine the final phenotype prediction from DNA [8]. These predictions were compared with actual data to assess model accuracy, categorizing matches as either correct or incorrect (Supplementary Table S4). Since the final prediction could involve more than one pigmentation (e.g., black/dark brown), a correct match was defined when at least one of the predicted colors was included in the actual color. An incorrect match was assigned when none of the predictive colors were found in the actual pigmentation. Due to incomplete genetic profiles (using 20 out of 22 of the established SNPs), our results here show an AUC of 0.813 for blond hair with an AUC loss of 0.003. For brown hair, the AUC is 0.741, with an AUC loss of 0.002. The AUC for red hair is 0.929 with an AUC loss of 0.013, and for black hair, the AUC is 0.859 with an AUC loss of 0.001.

Success of predicting hair pigmentation is detailed in Table 4, showing 43.24% accuracy for blond, 0% for red, 84.90% for brown, and 44.33% for black hair. The negative prediction results for red hair should not be considered, as it is based on data from only one participant and, thus, is not representative. Notably, a great percentage of individuals with blond (54.05%) and black hair (51.55%) were incorrectly assigned to brown/dark-brown hair (Table 4).

In the case of the blond category, our results align with conclusions drawn by other studies that attribute the lack of precision in predicting light hair colors to age-dependent hair darkening [1,8,41]. It is frequent for individuals to transition from blond hair in childhood to progressively darker hair in adulthood. Furthermore, subjective perceptions of hair pigmentation also play a role, making it difficult to differentiate between dark blond and light brown hair. Consequently, a considerable number of individuals misclassified as having brown hair might have been accurately categorized as blond with a different approach. To address these issues, one potential solution could be to include questions about age-related hair color changes during data collection [1]. However, this would not resolve the subjectivity involved in distinguishing between dark blond and light brown in certain cases. This same issue likely impacts the brown hair category, with most of the incorrect matches (11.43%) attributed to blond/dark-blond misclassifications. Future DNA-based prediction efforts should consider quantitative measures of hair color, which might improve performance and reduce interpretation challenges [1,26]. Meanwhile, when interpreting predictions for blond and brown hair within our population, it would be advisable to also consider the possibility of light brown and dark blond hair, respectively.

On the other hand, a considerable proportion of black-hair individuals (51.55%) predicted that they have brown/dark-brown hair, which may be attributed to the actual data since distinguishing between dark brown and black hair can be complicated in some cases. Subjectivity may also be an influencing factor in this context. To mitigate this challenge, and while future DNA-based prediction methods incorporating quantitative hair color are still under development, we propose a revised classification system for populations with predominantly dark hair, such as our Spanish population. The suggested categories would be blond, red, light brown, and a combined category of dark brown/black. Based on our population under study, merging dark brown and black categories may enhance prediction performance without significantly compromising individual descriptions. However, this approach may not be applicable to all populations, and further investigation is needed before making broader generalizations.

Consequently, the overall prediction performance metrics of the model were suboptimal, with AUC values of 0.336 for blond, 0.508 for red, 0.339 for brown, and 0.278 for black (Table 5), which markedly deviate from those reported by the HIrisPlex model: 0.80, 0.92, 0.72, and 0.83 for blond, red, brown and black hair, respectively [48]. Our low AUC values are the consequence of the model performance, which, despite having strong specificity for blond (89.50%) and black hair (100%), showed weak sensitivity for these categories (43.24% and 44.33%, respectively). Conversely, brown hair exhibited low specificity (47.41%) but high sensitivity (84.90%). These metrics reflect the high proportion of blond- and black-haired individuals being misclassified as brown-haired. These results suggest that, in our population, when the model predicts blond or black hair, it is highly likely to be accurate. Nevertheless, when predicting brown hair, although it is likely to be correct, a potential range of hair pigmentation from blond to black must be considered. Additionally, the small sample size for blond, red, and black hair may have contributed to these results.

Following the suboptimal performance observed in our dataset, particularly concerning blond and black hair pigmentation, we attempted to recalibrate the HIrisPlex model. Utilizing 20 of its 22 established markers for hair pigmentation prediction (*MC1R* N29insA and *MC1R* rs2228479 were excluded because of missing results) as input, we customized the model to our study population to assess potential enhancements.

In the MLR model, red hair was not considered because of its minimum representation (0.26%). The model obtained after the forward stepwise regression was based on 10 of the 20 initial SNPs (rs12913832, rs12203592, rs16891982, rs683, rs4959270, rs1805008, rs1805005, rs2378249, rs1805006, and rs28777), indicating that these genetic markers are the most informative for hair pigmentation in our population. Notably, like the iris color model, the marker *HERC2* rs12913832 demonstrated the strongest association. The AUC values for predicting the hair pigmentation categories after cross-validation were 0.663 for blond, 0.707 for brown, and 0.697 for black (Figure 3). These results indicate a considerable improvement in the prediction performance for all three categories compared to our previous outcomes for the same population using the HIrisPlex model (Table 5). This improvement may arise from the simplified nature of our model, which categorizes hair color into three broad groups, as opposed to the HIrisPlex model’s four categories, which include additional nuances such as dark blond and dark brown.

These findings, together with the results obtained using the original HIrisPlex model for hair pigmentation, suggest that although some less informative SNPs in the HIrisPlex model may introduce some noise into the predictions for our population, there remains a notable difficulty in distinguishing between certain shades of dark or light hair. This observation supports the necessity for future DNA-based prediction studies to move from qualitative to quantitative assessments of hair color. Such a shift could enhance performance and help mitigate interpretation challenges currently faced.

Similar to our approach with iris pigmentation, we tried to replicate hair color prediction using the online HIrisPlex-S tool, utilizing data solely from the ten significant SNPs (*HERC2* rs12913832, *IRF4* rs12203592, *SLC45A2* rs16891982, *TYRP1* rs683, *EXOC2* rs4959270, *MC1R* rs1805008, *MC1R* rs1805005, *ASIP/PIGU* rs2378249, *MC1R* rs1805006, and *SLC45A2* rs28777). However, this attempt was unsuccessful because the prediction model required data from three additional *MC1R* markers (*MC1R* rs11547464, *MC1R* rs1805007, and *MC1R* rs1805009) to function effectively, as specified by the guidelines of the HIrisPlex-S tool.

#### 3.2.3. Skin Pigmentation

The HIrisPlex-S system, which includes five categories (dark-black, dark, intermediate, pale, and very pale), was used to classify our skin pigmentation data [9]. When examining the distribution of skin color in our population (Figure 1 and Figure 2), we observed that the majority of individuals were classified as having pale (53.43%) and intermediate (39.71%) skin tones, while dark skin exhibited very low representation (6.86%). No representation was observed for the very pale and dark-black skin categories.

For skin pigmentation prediction, 33 out of the 36 SNPs specifically established by the HIrisPlex-S for skin color (*MC1R* rs1805007, *MC1R* rs1805008, *MC1R* rs11547464, *MC1R* rs885479, *MC1R* rs1805006, *MC1R* rs1110400, *IRF4* rs12203592, *OCA2* rs1800407, *SLC45A2* rs16891982, *SLC45A2* rs28777, *HERC2* rs12913832, *TYR* rs1042602, *TYR* rs1393350, *PIGU* rs2378249, *LOC105370627* rs12896399, *SLC24A4* rs2402130, *TYRP1* rs683, *KITLG* rs12821256, *ANKRD11* rs3114908, *BNC2* rs10756819, *SLC24A4* rs17128291, *HERC2* rs2238289, *HERC2* rs1129038, *HERC2* rs1667394, *TYR* rs1126809, *OCA2* rs1470608, *OCA2* rs12441727, *OCA2* rs1545397, *SLC24A5* rs1426654, *ASIP* rs6119471, *RALY* rs6059655, *MC1R* rs3212355, and *DEF8* rs8051733) were used. Due to incomplete genetic profiles (33 out of 36 of the established SNPs were used), our results here presented show an AUC of 0.830 for very pale skin with an AUC loss of 0.007 and an AUC of 0.763 with an AUC loss of −3.982E−04 for pale skin. For intermediate skin, the AUC is 0.783, with an AUC loss of 0.010. The AUC for dark skin is 0.981 with an AUC loss of −4.604 × 10^−4^, and for dark-black skin, the AUC is 0.993 with an AUC loss of −2.417 × 10^−4^.

To determine the most likely skin pigmentation using the HIrisPlex-S model, the predicted skin color category with the highest probability value was selected. However, the influence of the second highest probability value was also considered if it was deemed significant, as previously described [9]. Moreover, predictions were classified as undefined when there was any clear dominant category, meaning all probability values were below the 0.5 threshold. In our strict approach, final phenotype predictions were generated by selecting the predicted category that exceeded the 0.5 threshold and had the highest probability. These predictions were then compared with the actual data to assess performance, considering a correct match when both the predicted and actual data were the same or an incorrect match when the predictive color differed from the actual data. Cases where the predictions were undefined were also considered undefined matches (Supplementary Table S4).

Nevertheless, in accordance with the HIrisPlex-S guidelines, which recommend considering the influence of the second-highest probability category, a more flexible approach was adopted. In this approach, final phenotype predictions were generated considering not only the highest probability category but also other tones that might be contributing. Since the final prediction can involve more than one pigmentation (e.g., intermediate to pale), a correct match was defined as any case where at least one of the predicted colors matched the actual one. An incorrect match was assigned when none of the predictive colors corresponded to the actual pigmentation. Similar to the initial approach, cases where the predictions were undefined were also categorized as undefined matches (Supplementary Table S4).

The accuracy of predicting skin pigmentation was considerably low across both methods, with rates of 39.29% and 53.57% for dark, 67.90% and 70.37% for intermediate, and 0% and 0.92% for pale skin, according to the strict and flexible approach, respectively (Table 6 and Table 7). Undefined cases were more common in intermediate (14.20%) and pale skin (14.68%) compared to dark skin (3.57%). Notably, a significant percentage of individuals with dark (39.29%) and pale skin (75.23%) were incorrectly assigned to the intermediate pigmentation category. Our high percentage of incorrect predictions for pale skin is consistent with the lower accuracy of the HIrisPlex-S system for light skin categories compared to dark skin categories [9], supporting the need to find more predictive markers for light skin [26].

Inaccuracies in color skin prediction could also result from a combination of external factors, such as room lighting or the time of year when the information was collected, which can affect the selected category for each individual. The intermediate skin color often appears different in winter compared to summer. Additionally, similar to iris and hair, subjective perception of skin pigmentation can complicate the differentiation between categories, although we attempted to mitigate this by using the evaluations of three independent individuals. One possible strategy to reduce these limitations could involve employing spectrophotometry techniques to objectively measure skin color, preferably in areas not directly exposed to sunlight. Nevertheless, the considerable diversity in skin tones adds complexity to interpreting prediction results. Since predictions often involve a mix of different categories, cases may arise where the final prediction is a lighter or darker intermediate. It can be challenging to clearly distinguish between lighter intermediate and pale or darker intermediate and dark. Given the need for categorization, certain cases deemed incorrect may actually align with the scenarios described earlier and could be reclassified as correct under a different approach. As discussed with eye and hair predictions, future DNA-based prediction efforts should move to quantitative measures of skin color, which might enhance accuracy and help overcome interpretative challenges [1,26].

Despite the modest improvement with the flexible approach, the overall prediction performance metrics of the model were calculated based on this method as it yielded slightly better results. AUC values for predicting different pigmentation categories are presented in Table 8 (0.722 for dark, 0.501 for intermediate, and 0.495 for pale skin). In comparison to the values reported in the HIrisPlex-S [9], our results are considerably lower across all categories (0.88 for dark, 0.73 for intermediate, and 0.72 for light skin). These differences can be attributed to the sample size in both studies, which is considerably smaller in our case, especially in the dark and pale categories, as well as the lack of complete genetic profiles, which can reduce model performance. Additionally, external factors previously discussed may also contribute to the disparity in results. The large number of individuals incorrectly predicted as having intermediate skin is reflected in the low specificity obtained for this category (17.84%) despite its strong sensitivity (82.01%). On the contrary, specificity values were particularly high for dark (88.92%) and pale skin (100%), while sensitivity was lower (55.56% for dark and 1.08% for pale skin). These results suggest that, in our population, when the model predicts dark or pale skin, it is highly likely to be accurate. Nevertheless, when predicting intermediate skin, a potential range from pale to dark must be considered.

In response to the less-than-ideal performance observed in our dataset, especially concerning dark and pale skin tones, we sought to refine the HIrisPlex-S model in our population. We utilized 33 out of the 36 established markers in the HIrisPlex-S assay as input for predicting skin pigmentation (excluding *MC1R* rs2228479, *HERC2* rs6497292, and *OCA2* rs1800414 because of missing results). With this customization, we attempted to optimize the model for our specific study population and potentially enhance its predictive performance.

The forward stepwise MLR model obtained was based on 11 of the 33 initial SNPs (rs16891982, rs12913832, rs6119471, rs4959270, rs1805008, rs683, rs10756819, rs1545397, rs1110400, rs12896399, and rs885479), indicating that these genetic markers are the most informative for skin pigmentation in our population. The AUC values achieved for skin pigmentation prediction after cross-validation were 0.621 for dark, 0.593 for pale, and 0.556 for intermediate skin (Figure 3). These results only show a slight enhancement in predicting intermediate and pale skin tones, decreasing the performance for the dark category compared to previous results obtained from the same population using the HIrisPlex-S system (Table 8). Considering all the results, we suggest that the need for additional predictive SNPs for light skin might not be the only issue; external factors affecting data collection also play a significant role and should be taken into account.

In eye, hair, and skin color prediction, a recurring challenge lies in determining the accuracy of predictions for complex phenotypes. This difficulty is particularly evident in the intermediate category for eye pigmentation, where, despite applying more flexible criteria, there are still cases where the probability values obtained may indicate ambiguous categories that are difficult to interpret (e.g., intermediate-blue or intermediate-brown phenotypes). The complexity further increases in hair color prediction, not only because of the four established categories but also because of the inclusion of two additional categories that refine pigmentation shades. Categories like dark blond and light brown can overlap, making it difficult to definitively classify a prediction as correct or incorrect, often leaving interpretation up to the discretion of the individual analyzing the results. Skin color prediction presents the greatest challenge because of the broad diversity of skin tones. Predictions frequently span multiple categories, leading to ambiguous outcomes (e.g., lighter intermediate or dark-to-intermediate) and complicating the assessment of prediction accuracy. Although categorical classifications are valuable for describing an individual’s appearance and aiding in identification, they also introduce a degree of subjectivity. This subjectivity can influence not only individuals’ perception of their own pigmentation, which can impact data collection and subsequent comparison with predictions but also the interpretation of results. In ambiguous cases, one person might consider a match incorrect, and another might deem it correct. This challenge arose during our sample collection, despite our efforts to minimize subjectivity by requiring agreement among multiple researchers. While we aimed for objectivity, a certain degree of subjectivity was inevitable. As noted earlier, future studies would benefit from incorporating more objective methods, such as spectrophotometry.

These observations underscore the ongoing challenges in making definitive predictions, particularly in cases where, despite following guidelines meticulously, ambiguous outcomes or undefined predictions persist. In light of these issues, many researchers suggest transitioning from categorical systems to quantitative measures of color [1,26]. Such a shift could simplify the interpretation of predictions and enhance the accuracy of comparisons with actual phenotypes.

## 4. Conclusions

Our study aimed to evaluate the performance of the HIrisPlex-S system in a Spanish population. We calculated various metrics, including accuracy, AUC, sensitivity, specificity, PPV, and NPV, observing favorable outcomes for blue and brown eyes, brown hair, and intermediate skin, with accuracy rates ranging from 70% to 97%. However, we encountered issues with the remaining categories, which involved more complex phenotypes. Particularly in eye color prediction, despite applying more flexible criteria, most cases of intermediate eye pigmentation were still predicted inaccurately. Similarly, inaccuracies were observed in hair pigmentation, where distinguishing between dark brown and black hair, as well as dark blond and light brown, posed challenges. These difficulties were even more pronounced in skin pigmentation prediction, with significant misclassification observed, particularly among individuals with dark and pale skin.

One limitation of our study is the limited sample size, which also results in some pigmentation categories being underrepresented. Additionally, as previously mentioned, certain genetic markers reported missing results, leading to predictions based on fewer markers than those included in the HIrisPlex-S models, specifically 20 out of the established 22 SNPs for hair color prediction and 30 out of the 36 markers used for skin color prediction. While complete genetic profiles are not mandatory for obtaining predictions, their absence might reduce prediction accuracy. Although the AUC loss values observed in our case were minimal, this factor should still be considered potentially influential. Nonetheless, we encountered significant challenges in interpreting the final predictions within this population, which may not be entirely attributable to our limitations. We have proposed some potential solutions which may offer valuable insights for similar populations. However, to draw solid conclusions and make broader generalizations, further validation in larger and more diverse populations is needed.

## Figures and Tables

**Table 1:** Prediction success percentage for each eye color category after using the HIrisPlex-S model and comparing the results with the actual data following the strict approach. NA stands for Not Assigned.

Eye Pigmentation Predicted by HIrisPlex-S	Correct	Incorrect		Uncertain	NA	N
Blue	93.75%	3.13%		3.33% ^1^	-	32
		Intermediate	Brown			
		3.13%	0%			
Intermediate	10.47%	66.28%		23.26% ^2^	-	86
		Blue	Brown			
		15.12%	51.16%			
Brown	88.08%	1.92%		9.23% ^3^	0.77%	260
		Blue	Intermediate			
		0.38%	1.54%			
Total						378

^1^ All 3.33% represent possible intermediate-brown eyes. ^2^ Secondly, 23.26% is divided into 20.93%, which represents possible intermediate-brown eyes, and 2.33%, which represents possible intermediate-blue eyes. ^3^ Finally, 9.23% is divided into 8.85%, which represents possible intermediate-brown eyes, and 0.38%, which represents possible intermediate-blue eyes.

**Table 2:** Prediction success percentage for each eye color category after using the HIrisPlex-S model and compare the results with the actual data following the flexible approach. NA stands for Not Assigned.

Eye Pigmentation Predicted by HIrisPlex-S	Correct	Incorrect	NA	N
Blue	93.75%	6.25%	-	32
Intermediate	33.72%	66.28%	-	86
Brown	96.92%	2.31%	0.77%	260
Total				378

**Table 3:** Summary statistics (accuracy, AUC, sensitivity, specificity, PPV, and NPV) for eye pigmentation prediction in our population under study. AUC stands for Area Under the Curve, PPV for Positive Predictive Value, and NPV for Negative Predictive Value.

Eye Pigmentation Predicted by HirisPlex-S	Accuracy	AUC	Sensitivity	Specificity	PPV	NPV
Blue	93.75%	0.948	93.75%	95.93%	68.18%	99.40%
Intermediate	33.72%	0.344	33.72%	97.59%	80.56%	83.24%
Brown	96.92%	0.802	97.67%	62.71%	85.14%	92.50%

**Table 4:** Prediction success percentage for each hair color category after using the HIrisPlex-S model and compare the results with the actual data.

Hair Pigmentation Predicted by HIrisPlex-S	Correct	Incorrect			N
Blond	43.24%	56.76%			37
		Red	D/L-Brown		
		2.70%	54.05%		
Red	0%	100%			1
		D/L-Brown			
		100%			
Brown	96.33%	3.67%			245
		Blond	Red		
		2.45%	1.22%		
Black	44.33%	55.67%			97
		Blond/D-Blond/L-Brown	Red	D/L-Brown	
		2.06%	2.06%	51.55%	
Total					380

**Table 5:** Summary statistics (accuracy, AUC, sensitivity, specificity, PPV, and NPV) for hair pigmentation prediction in our population under study. AUC stands for Area Under the Curve, PPV for Positive Predictive Value, and NPV for Negative Predictive Value.

Hair Pigmentation Predicted by HirisPlex-S	Accuracy	AUC	Sensitivity	Specificity	PPV	NPV
Blond	43.24%	0.336	43.24%	89.50%	30.77%	93.60%
Red	0%	0.508	0%	98.42%	0%	99.73%
Brown	96.33%	0.339	84.90%	47.41%	74.55%	63.37%
Black	44.33%	0.278	44.33%	100%	100%	83.98%

**Table 6:** Prediction success percentage for each skin color category after using the HIrisPlex-S model and compare the results with the actual data following the strict approach.

Skin Pigmentation Predicted by HIrisPlex-S	Correct	Incorrect				Undefined	N
Dark	39.29%	57.14%				3.57%	28
		Intermediate	Dark-black				
		50%	7.14%				
Intermediate	67.90%	17.90%				14.20%	162
		Pale	Dark	Dark-black			
		1.23%	12.96%	3.70%			
Pale	0%	85.32%				14.68%	218
		Very pale	Intermediate	Dark	Dark-black		
		0.92%	75.23%	5.50%	3.67%		
Total							408

**Table 7:** Prediction success percentage for each skin color category after using the HIrisPlex-S model and compare the results with the actual data following the flexible approach.

Skin Pigmentation Predicted by HIrisPlex-S	Correct	Incorrect			Undefined	N
Dark	53.57%	42.86%			3.57%	28
		Intermediate	Dark-black			
		39.29%	3.57%			
Intermediate	70.37%	15.43%			14.20%	162
		Dark	Dark-black			
		12.96%	2.47%			
Pale	0.92%	85.32%			14.68%	218
		Intermediate	Dark	Dark-black		
		75.23%	5.50%	3.67%		
Total						408

**Table 8:** Summary statistics (accuracy, AUC, sensitivity, specificity, PPV, and NPV) for skin pigmentation prediction in our population under study. AUC stands for Area Under the Curve, PPV stands for Positive Predictive Value, and NPV stands for Negative Predictive Value.

Skin Pigmentation Predicted by HirisPlex-S	Accuracy	AUC	Sensitivity	Specificity	PPV	NPV
Dark	53.57%	0.722	55.56%	88.92%	29.41%	96.01%
Intermediate	70.37%	0.501	82.01%	17.84%	39.45%	60.32%
Pale	0.92%	0.495	1.08%	100%	100%	47.43%

## Data Availability

In view of the highly identifiable nature of facial and genomic information, a more conservative approach was adopted in recruiting individuals. Distributing the raw data from this collection widely would be a legal and ethical violation of the informed consent obtained from participants. This restriction is not due to any personal or commercial interest. Further information is available from MMP on request.

## Supplementary Materials

The following supporting information can be downloaded at https://www.mdpi.com/article/10.3390/genes15101330/s1, Table S1: summary of the SNPs analyzed; Table S2: amplicon size, sequences, and concentration of SNaPshot primers used in first amplification and minisequencing; Table S3: allele and genotype frequencies in SNPs analyzed; Table S4: Results of phenotypes obtained after applying the HIrisPlex-S prediction tool and comparison with the associated data.

## References

[1] Kayser M. (2015). Forensic DNA Phenotyping: Predicting human appearance from crime scene material for investigative purposes. Forensic Sci. Int. Genet..

[2] Katsara M.-A., Nothnagel M. (2019). True colors: A literature review on the spatial distribution of eye and hair pigmentation. Forensic Sci. Int. Genet..

[3] Frudakis T., Terravainen T., Thomas M. (2007). Multilocus OCA2 genotypes specify human iris colors. Hum. Genet..

[4] Allwood J.S., Harbison S. (2013). SNP model development for the prediction of eye colour in New Zealand. Forensic Sci. Int. Genet..

[5] Maroñas O., Phillips C., Söchtig J., Gomez-Tato A., Cruz R., Alvarez-Dios J., de Cal M.C., Ruiz Y., Fondevila M., Carracedo Á. (2014). Development of a forensic skin colour predictive test. Forensic Sci. Int. Genet..

[6] Ruiz Y., Phillips C., Gomez-Tato A., Alvarez-Dios J., de Cal M.C., Cruz R., Maroñas O., Söchtig J., Fondevila M., Rodriguez-Cid M. (2013). Further development of forensic eye color predictive tests. Forensic Sci. Int. Genet..

[7] Valenzuela R.K., Henderson M.S., Walsh M.H., Garrison N.A., Kelch J.T., Cohen-Barak O., Erickson D.T., Meaney F.J., Walsh J.B., Cheng K.C. (2010). Predicting Phenotype from Genotype: Normal Pigmentation. J. Forensic Sci..

[8] Walsh S., Liu F., Wollstein A., Kovatsi L., Ralf A., Kosiniak-Kamysz A., Branicki W., Kayser M. (2013). The HIrisPlex system for simultaneous prediction of hair and eye colour from DNA. Forensic Sci. Int. Genet..

[9] Chaitanya L., Breslin K., Zuñiga S., Wirken L., Pośpiech E., Kukla-Bartoszek M., Sijen T., de Knijff P., Liu F., Branicki W. (2018). The HIrisPlex-S system for eye, hair and skin colour prediction from DNA: Introduction and forensic developmental validation. Forensic Sci. Int. Genet..

[10] Walsh S., Liu F., Ballantyne K.N., van Oven M., Lao O., Kayser M. (2011). IrisPlex: A sensitive DNA tool for accurate prediction of blue and brown eye colour in the absence of ancestry information. Forensic Sci. Int. Genet..

[11] Sulem P., Gudbjartsson D.F., Stacey S.N., Helgason A., Rafnar T., Magnusson K.P., Manolescu A., Karason A., Palsson A., Thorleifsson G. (2007). Genetic determinants of hair, eye and skin pigmentation in Europeans. Nat. Genet..

[12] Kayser M., Liu F., Janssens A.C.J., Rivadeneira F., Lao O., van Duijn K., Vermeulen M., Arp P., Jhamai M.M., van Ijcken W.F. (2008). Three genome-wide association studies and a linkage analysis identify HERC2 as a human iris color gene. Am. J. Hum. Genet..

[13] Liu F., van Duijn K., Vingerling J.R., Hofman A., Uitterlinden A.G., Janssens A.C.J., Kayser M. (2009). Eye color and the prediction of complex phenotypes from genotypes. Curr. Biol..

[14] Mengel-From J., Børsting C., Sanchez J.J., Eiberg H., Morling N. (2010). Human eye colour and HERC2, OCA2 and MATP. Forensic Sci. Int. Genet..

[15] Spichenok O., Budimlija Z.M., Mitchell A.A., Jenny A., Kovacevic L., Marjanovic D., Caragine T., Prinz M., Wurmbach E. (2011). Prediction of eye and skin color in diverse populations using seven SNPs. Forensic Sci. Int. Genet..

[16] Pneuman A., Budimlija Z.M., Caragine T., Prinz M., Wurmbach E. (2012). Verification of eye and skin color predictors in various populations. Leg. Med..

[17] Hart K.L., Kimura S.L., Mushailov V., Budimlija Z.M., Prinz M., Wurmbach E. (2013). Improved eye- and skin-color prediction based on 8 SNPs. Croat. Med. J..

[18] Liu F., Wollstein A., Hysi P.G., Ankra-Badu G.A., Spector T.D., Park D., Zhu G., Larsson M., Duffy D.L., Montgomery G.W. (2010). Digital quantification of human eye color highlights genetic association of three new loci. PLoS Genet..

[19] Freire-Aradas A., Ruiz Y., Phillips C., Maroñas O., Söchtig J., Tato A.G., Dios J., de Cal M.C., Silbiger V., Luchessi A. (2014). Exploring iris colour prediction and ancestry inference in admixed populations of South America. Forensic Sci. Int. Genet..

[20] Branicki W., Brudnik U., Kupiec T., Wolañska-Nowak P., Wojas-Pelc A. (2007). Determination of Phenotype Associated SNPs in the MC1R Gene. J. Forensic Sci..

[21] Draus-Barini J., Walsh S., Pośpiech E., Kupiec T., Głąb H., Branicki W., Kayser M. (2013). Bona fide colour: DNA prediction of human eye and hair colour from ancient and contemporary skeletal remains. Investig. Genet..

[22] Walsh S., Lindenbergh A., Zuniga S.B., Sijen T., de Knijff P., Kayser M., Ballantyne K.N. (2011). Developmental validation of the IrisPlex system: Determination of blue and brown iris colour for forensic intelligence. Forensic Sci. Int. Genet..

[23] Yun L., Gu Y., Rajeevan H., Kidd K.K. (2014). Application of six IrisPlex SNPs and comparison of two eye color prediction systems in diverse Eurasia populations. Int. J. Leg. Med..

[24] Dembinski G.M., Picard C.J. (2014). Evaluation of the IrisPlex DNA-based eye color prediction assay in a United States population. Forensic Sci. Int. Genet..

[25] Grimes E.A., Noake P.J., Dixon L., Urquhart A. (2001). Sequence polymorphism in the human melanocortin 1 receptor gene as an indicator of the red hair phenotype. Forensic Sci. Int..

[26] Kayser M., Branicki W., Parson W., Phillips C. (2023). Recent advances in Forensic DNA Phenotyping of appearance, ancestry and age. Forensic Sci. Int. Genet..

[27] Visconti A., Duffy D.L., Liu F., Zhu G., Wu W., Chen Y., Hysi P.G., Zeng C., Sanna M., Iles M.M. (2018). Genome-wide association study in 176,678 Europeans reveals genetic loci for tanning response to sun exposure. Nat. Commun..

[28] Crawford N.G., Kelly D.E., Hansen M.E.B., Beltrame M.H., Fan S., Bowman S.L., Jewett E., Ranciaro A., Thompson S., Lo Y. (2017). Loci associated with skin pigmentation identified in African populations. Science.

[29] Jonnalagadda M., Faizan M.A., Ozarkar S., Ashma R., Kulkarni S., Norton H.L., Parra E. (2019). A Genome-Wide Association Study of Skin and Iris Pigmentation among Individuals of South Asian Ancestry. Genome Biol. Evol..

[30] Samuels B.D., Aho R., Brinkley J.F., Bugacov A., Feingold E., Fisher S., Gonzalez-Reiche A.S., Hacia J.G., Hallgrimsson B., Hansen K. (2020). FaceBase 3: Analytical tools and FAIR resources for craniofacial and dental research. Development.

[31] Weinberg S.M., Raffensperger Z.D., Kesterke M.J., Heike C.L., Cunningham M.L., Hecht J.T., Kau C.H., Murray J.C., Wehby G.L., Moreno L.M. (2016). The 3D Facial Norms Database: Part 1. A Web-Based Craniofacial Anthropometric and Image Repository for the Clinical and Research Community. Cleft Palate-Craniofacial. J..

[32] Heike C.L., Upson K., Stuhaug E., Weinberg S.M. (2010). 3D digital stereophotogrammetry: A practical guide to facial image acquisition. Head Face Med..

[33] Miller S.A., Dykes D.D., Polesky H.F. (1988). A simple salting out procedure for extracting DNA from human nucleated cells. Nucleic Acids Res..

[34] Marshall O.J. (2004). PerlPrimer: Cross-platform, graphical primer design for standard, bisulphite and real-time PCR. Bioinformatics.

[35] Vallone P.M., Butler J.M. (2004). AutoDimer: A screening tool for primer-dimer and hairpin structures. BioTechniques.

[36] Ye J., Coulouris G., Zaretskaya I., Cutcutache I., Rozen S., Madden T.L. (2012). Primer-BLAST: A tool to design target-specific primers for polymerase chain reaction. BMC Bioinform..

[37] Excoffier L., Lischer H.E.L. (2010). Arlequin suite ver 3.5: A new series of programs to perform population genetics analyses under Linux and Windows. Mol. Ecol. Resour..

[38] IBM Corp (2021). IBM SPSS Statistics for Windows, Version 28.0.

[39] The 1000 Genomes Project Consortium (2015). A global reference for human genetic variation. Nature.

[40] Walsh S., Wollstein A., Liu F., Chakravarthy U., Rahu M., Seland J.H., Soubrane G., Tomazzoli L., Topouzis F., Vingerling J.R. (2012). DNA-based eye colour prediction across Europe with the IrisPlex system. Forensic Sci. Int. Genet..

[41] O I.S., Simsek S.Z., Filoglu G., Bulbul O. (2022). Predicting Eye and Hair Color in a Turkish Population Using the HIrisPlex System. Genes.

[42] Kastelic V., Pośpiech E., Draus-Barini J., Branicki W., Drobnič K. (2013). Prediction of eye color in the Slovenian population using the IrisPlex SNPs. Croat. Med. J..

[43] Salvoro C., Faccinetto C., Zucchelli L., Porto M., Marino A., Occhi G., de Los Campos G., Vazza G. (2019). Performance of four models for eye color prediction in an Italian population sample. Forensic Sci. Int. Genet..

[44] Chaitanya L., Walsh S., Andersen J.D., Ansell R., Ballantyne K., Ballard D., Banemann R., Bauer C.M., Bento A.M., Brisighelli F. (2014). Collaborative EDNAP exercise on the IrisPlex system for DNA-based prediction of human eye colour. Forensic Sci. Int. Genet..

[45] Pietroni C., Andersen J.D., Johansen P., Andersen M.M., Harder S., Paulsen R., Børsting C., Morling N. (2014). The effect of gender on eye colour variation in European populations and an evaluation of the IrisPlex prediction model. Forensic Sci. Int. Genet..

[46] Eiberg H., Troelsen J., Nielsen M., Mikkelsen A., Mengel-From J., Kjaer K.W., Hansen L. (2008). Blue eye color in humans may be caused by a perfectly associated founder mutation in a regulatory element located within the HERC2 gene inhibiting OCA2 expression. Hum. Genet..

[47] Sturm R.A., Duffy D.L., Zhao Z.Z., Leite F.P., Stark M.S., Hayward N.K., Martin N.G., Montgomery G.W. (2008). A single SNP in an evolutionary conserved region within intron 86 of the HERC2 gene determines human blue-brown eye color. Am. J. Hum. Genet..

[48] Walsh S., Chaitanya L., Clarisse L., Wirken L., Draus-Barini J., Kovatsi L., Maeda H., Ishikawa T., Sijen T., de Knijff P. (2014). Developmental validation of the HIrisPlex system: DNA-based eye and hair colour prediction for forensic and anthropological usage. Forensic Sci. Int. Genet..

